# Polarization of an electroactive functional film on titanium for inducing osteogenic differentiation

**DOI:** 10.1038/srep35512

**Published:** 2016-10-20

**Authors:** Zhengnan Zhou, Weiping Li, Tianrui He, Lei Qian, Guoxin Tan, Chengyun Ning

**Affiliations:** 1 School of Materials Science and Engineering, South China University of Technology, Guangzhou 510641, China; 2 Guangdong Key Laboratory of Biomedical Sciences and Engineering, South China University of Technology, Guangzhou 510006, China; 3 Institute of Chemical Engineering and Light Industry, Guangdong University of Technology, Guangzhou 510006, China

## Abstract

To enhance the surface bioactivity of titanium (Ti) prostheses, an electroactive polyvinylidene fluoride (PVDF) film was prepared on a Ti substrate to provide a mimetic of the electrical microenvironment, which facilitated the performance of cell functions. The results of cell proliferation and differentiation assays indicated that polarization of the PVDF-Ti (PTi) altered its surface charge, thus inducing adhesion, proliferation and osteogenic differentiation of cells. The polarized PVDF-Ti (PPTi) may therefore find applications in bone regeneration.

Biomedical titanium (Ti) and its alloys have been widely and clinically used in orthopaedic implants because they exhibit high machinability and favourable acceptability by human tissues under diverse circumstances^1^,^2^. However, Ti implants suffer from the weakness of the bioinert surface, which leads to failure of the implant. In orthopaedics, novel techniques have been employed, and numerous attempts have been made to enhance the surface activity of Ti prostheses, including modification of surface properties and coating with active layers^1^. Surface modifications have been implemented through sandblasting^3^, acid-etching^4^, micro-arc oxidation^5^ and plasma spraying of hydroxyapatite^6^,^7^ to improve the biological performance of Ti implants. The modified surfaces obtained using these methods exhibit good bioactivity, but show poor interfacial adhesion. Another problem is that these modified surfaces are merely passively tolerated by cells, which has negative effects on the performance of cells. Ideal surface coatings should actively provide an appropriate environment to facilitate cellular contact and signalling, allowing cells to perform their functions effectively.

Electroactive polymers are intriguing because they might provide a mimetic electrical microenvironment for cells in response to mechanical stimuli^8^, which is helpful for the induction of cell proliferation, assembly, and differentiation^9^,^10^. PVDF is a particularly suitable electroactive film used for the modification of Ti because of its favourable piezoelectricity, mechanical properties and biocompatibility^11^. It also mimics the endogenous electric potential, as the piezoelectric behaviour of bone and the electric potential of native bone have been demonstrated. Polarization of PVDF film generates surface charges and electric potential, which lead to stem cell adhesion and proliferation on the film and can induce cell osteogenic differentiation^12^,^13^,^14^. Zhang *et al*. have fabricated a nanocomposite piezoelectric membrane with PVDF and BaTiO_3_ to enhance bone regeneration through restoring physiological electric microenvironment^15^.

Here, we propose that an electroactive PVDF film on a Ti substrate will provide an appropriate electrical microenvironment, for inducing cell proliferation and osteogenic differentiation. In this study, an electroactive PVDF film on Ti (PVDF-Ti, PTi) was prepared via the tape-casting method. Scanning kelvin probe microscopy (SKPM) and atomic force microscope (AFM) were utilized to investigate the electric properties of the PTi. The effect of polarization of the PTi on the induction of osteogenic differentiation in bone marrow mesenchymal stem cells (BMSCs) was explored (Fig. 1). The experimental group and the control group were polarized PVDF-Ti (PPTi) and nonpolarized PVDF-Ti (NPTi), respectively.

## Results

### Characterization

The surface topography of the PTi was observed via SEM and AFM. The PPTi and NPTi were round and porous (Fig. S1(a,b)). The process of poling the PTi induced no significant differences in morphology or sample topography. Element energy dispersive spectrum analysis (EDS) confirmed the removal of organic solvents. PVDF is polymorphic and can form different crystalline structures, such as alpha, beta and gamma structures, depending on the crystallization conditions^16^. Fourier transform infrared (FTIR) spectroscopy is an appropriate tool for identification of the characteristic absorption band phases of PVDF. The attenuated total reflectance-fourier transform infrared (ATR-FTIR) spectra of the PPTi and NPTi are presented in Fig. S2(a). The PPTi and NPTi both exhibited prominent β-phase peaks at 836 cm^−1^ and 873 cm^−1^, with small α-phase peaks being observed at 762 cm^−1^ and 873 cm^−1^ 11,17,18. The fraction of the crystalline form in a crystal can be estimated from the absorbance of the characteristic peaks of all crystalline forms and their absorption coefficients^19^. The absorbance at 762 cm^−1^ (A_α_) and 836 cm^−1^ (A_β_) and the absorption coefficients of the α form (K_α_ = 6.1 × 10^4^ cm^2^/mol) and the β form (K_β_ = 7.7 × 10^4^ cm^2^/mol) can be used to calculate the mass fraction of the α form (Fα) in the crystal using Eq. 1 (assuming that the densities of the α and β crystallites are equal)^19^:


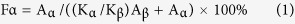


The relative fraction of the β-phase [F_β_] PVDF in the films was calculated. F_β_ of the PPTi and NPTi was 75.95% and 73.93% within the crystalline regions, respectively. The degree of crystallinity of the PPTi and NPTi was 48.5% and 45.3%, respectively, as calculated from the DSC results using the following equation Eq. 2 (Fig. S2(b))^17^,^20^.


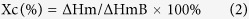


where ΔHm is the crystalline-phase melting enthalpy per gram of film from PTi; ΔHmB is the enthalpy value corresponding to the melting of 100% crystalline PVDF film (ΔHmB = 104.7 J/g).

X ray diffraction (XRD) analysis of the PPTi and NPTi revealed distinct peaks at 18.56° and 20.36° corresponding to crystal planes at (020) for α-phase PVDF and (110) for β-phase PVDF, respectively (Fig. S2(c))^11^,^19^,^21^.

### PFM results

Figure 2 shows the PFM amplitude and phase images of the samples within a 5 × 5 μm scan area. As demonstrated in Fig. 2(a1,b1), the PFM amplitude images confirmed that the β-phase regions exhibited piezoelectric properties. Compared with the NPTi, a higher piezoelectric response was observed in the PPTi, which exhibited a higher β-phase content within the crystalline region^22^,^23^. The phase images presented in Fig. 2(a2,b2) revealed distinct contrasts, which may indicate that the poling of the PTi was not complete. Contrast variations in the phase image were the result of the presence of a number of domains in the scan area^22^,^23^. Consistent with the calculations based on the FTIR analysis, the PPTi displayed a slightly higher proportion of polarized β-phase.

Theoretically, a PVDF polymer serves as an insulator of electricity, particularly in amorphous regions. When the sample is subjected to AC bias during AFM scanning, charge accumulation occurs on the surface^24^. Charge accumulation creates an electrostatic force between the tip and sample, which results in an artificial phase shift toward the positive voltage direction. The SKPM surface potential exhibited two distinct values (−930 ± 12 mV and −157 ± 8 mV) for the PPTi and NPTi. The SKPM and zeta potential analyses of the PPTi and NPTi revealed that the surface of the PPTi accumulated more negative charges (Fig. 3).

### Cell proliferation and differentiation

The proliferation and differentiation profiles of BMSCs cultured on the PPTi and NPTi are presented in Fig. 4. The proliferation of BMSCs was investigated using the CCK-8 assay. Figure 4(a) showed that the proliferation of BMSCs was greater on the PPTi than NPTi at 4 and 7 days. On day 1, cell proliferation on the NPTi was similar to that on the PPTi. Alkaline phosphatase (ALP) activity was examined to evaluate the osteogenic differentiation of BMSCs on the PPTi and NPTi after 7, 14 and 21 days of cell incubation. The ALP activity of the BMSCs cultured on the PPTi was slightly higher than in the cells cultured on the NPTi at 7 and 14 days (Fig. 4(b)). After 21 days, a significant difference was observed. This difference may be attributed to the increased negative charge of the PPTi. Therefore, the PPTi exhibited a good biocompatibility and osteogenic ability *in vitro*. These findings were further supported by the real-time polymerase chain reaction (RT-PCR) analysis of other osteogenic marker genes (ALP, collagen I and osteopontin) (Fig. 5), in which the cells on the PPTi exhibited significantly higher gene expression (*p < 0.05, **p < 0.01) than those on the NPTi after 14 days of incubation.

Representative scanning electron microscope (SEM) images of BMSCs fixed at specific time points were used to evaluate differences in cell morphology on the PPTi and NPTi (Fig. 6). Cells typically required 2–4 h to adhere to the TCPS surface but required up to 12 h for attachment on the PTi. Because our PTi samples were hydrophobic, the cells were weakly attached to the samples and may have been washed off due to the rinsing and dehydration procedures performed on day 1. Therefore, the assays were carried out on day 7. As shown in Fig. 6, the morphologies of BMSCs on the PPTi and NPTi were different. Spindle and triangular cells were observed on the PPTi, and more BMSCs exhibited larger filopodia on the PPTi.

## Discussion

Piezoelectric effects have been explored in bone-tissue regeneration since the first observation of these effects in bone. Piezoelectric films can induce *in vivo* formation of periosteal bone^25^. Electrical osteogenesis has been proved by X ray photographs and histological studies performed during a first series of implantations of piezoelectric and non-piezoelectric PVDF films. Ficat *et al*. had studied the osteogenic power of two kinds of PVDF films (monomorph PVDF films and bimorph PVDF films), and they attributed the formation of periosteal bone induced by piezoelectric PVDF films to a piezoelectric effect^26^. Previous studies of osteoblast-PVDF interactions have demonstrated that these interactions can be exploited clinically to promote tissue growth^13^,^27^. The different phase types of PVDF films can affect the adhesion and proliferation of cells in different ways^28^. Surface topography and surface wettability also greatly influence cell adhesion and proliferation. Charged surface promote osteoblast adhesion and proliferation. Thus, the combination of surface properties and piezoelectricity is a key factor in the induction of cell adhesion, proliferation and osteogenic differentiation on Ti with a layer of piezoelectric material.

**Figure 1:**
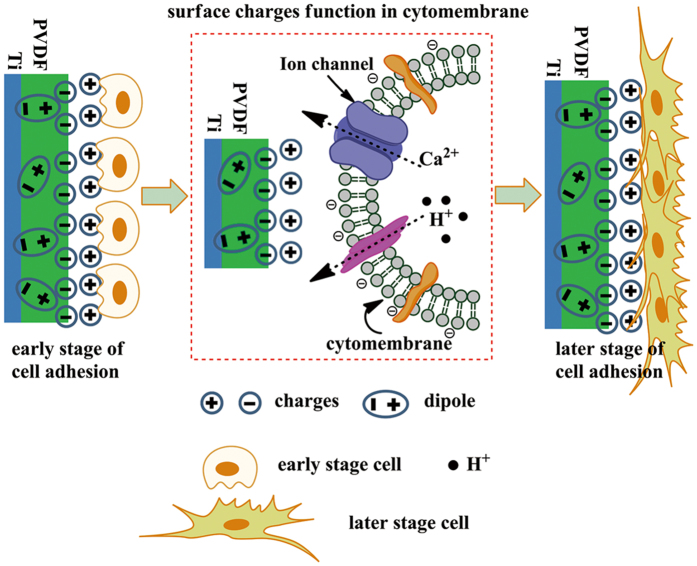
Schematic illustration of a potential mechanism for the enhanced cellular response of the PPTi. After polarization, the dipoles of the PTi become aligned, resulting in the distribution of negative charges on the surface. Among the ionic/biological constituents present in the growth medium, cations adhere strongly to the negatively charged surfaces due to electrostatic interactions between charged entities. The proteins and negatively charged cytomembrane are then attracted to one another. Consequently, the PPTi enhances cell adhesion and proliferation due to the presence of a greater surface charge compared with the NPTi.

**Figure 2:**
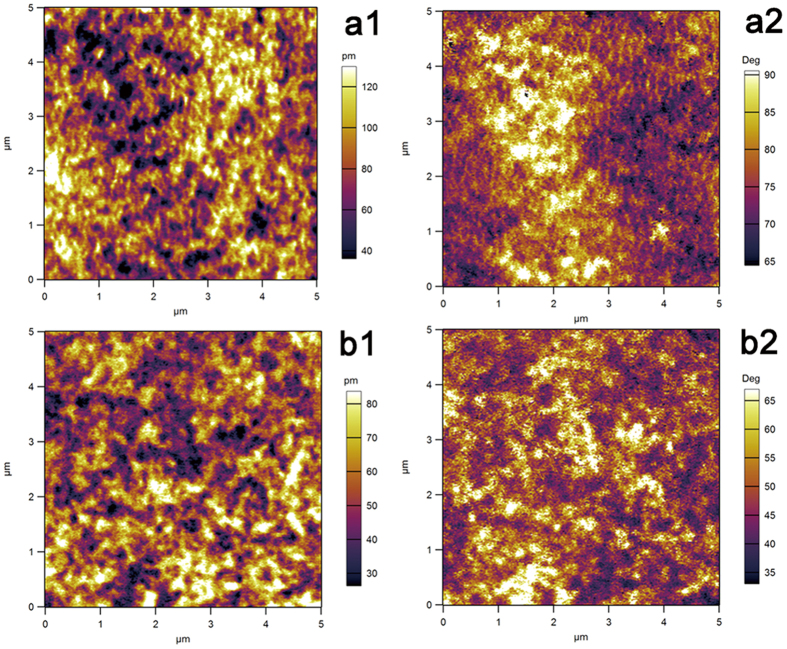
PFM measurements of the PPTi (**a**) and NPTi (**b**); PFM magnitude images are presented in a1 and b1, and phase images are presented in a2, and b2. In the magnitude images (**a1,b1**), bright regions indicate regions with a high piezo-response. The applied AC voltage was 10 V.

**Figure 3:**
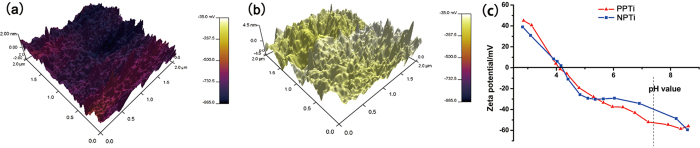
SKPM analysis of the surface potential of the PPTi (**a**) and NPTi (**b**). The 3D map combines the height and potential profiles and shows that the observed potential change is not due to a height change. This figure demonstrates that the surface potential of the PPTi is increased by polarization. (**c**) Results of zeta potential analysis for the PPTi and NPTi. Although the isoelectric points of the PPTi and NPTi were approximately equal, more negative charges were observed on the PPTi surface in a neutral solution environment.

**Figure 4:**
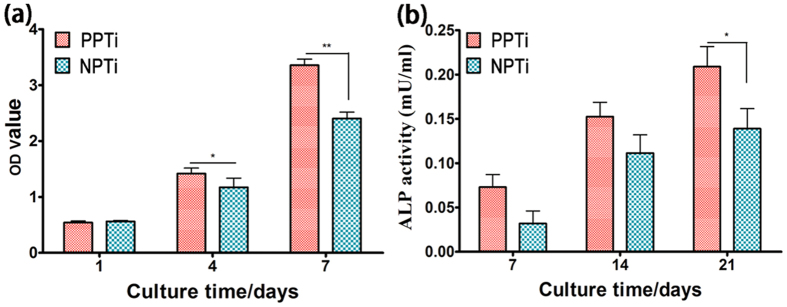
(**a**) Analysis of cell proliferation (CCK-8 assay) on the surface of the PPTi and NPTi after incubation with BMSCs for 1, 4 and 7 days (n = 4). A significant difference in cell proliferation was observed between the PPTi and NPTi at 4 days and 7 days, suggesting that the PPTi exhibits good biocompatibility and an improved cell proliferation ability. *p < 0.05, **p < 0.01 compared to the NPTi. (**b**) Osteogenic differentiation of BMSCs on the PPTi and NPTi as indicated by ALP activity after 7, 14 and21 days of culture in osteogenic media. Osteogenic cell differentiation was significantly greater on the PPTi at 21 days compared with the NPTi (*p < 0.05).

**Figure 5:**
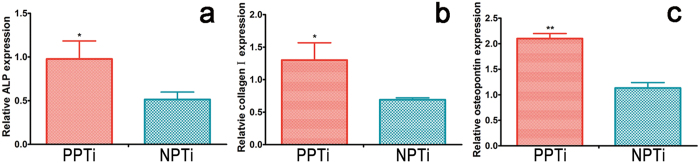
qRT-PCR analysis of osteogenic differentiation related gene expression on the surface of the PPTi and NPTi after incubation with BMSCs for 14 days (n = 3). (**a**) Relative ALP gene expression in BMSCs on different substrates after incubation for 14 days (n = 3). (**b**) Relative collagen I gene expression in BMSCs on different substrates after 14 days of culture (n = 3). (**c**) Relative osteopontin gene expression in BMSCs cultured on the PPTi and NPTi after incubation for 14 days. *p < 0.05, **p < 0.01 compared with NPTi. The results indicated that osteogenic differentiation-related gene expression in BMSCs was significantly greater on the PPTi at 14 days compared with the NPTi.

**Figure 6:**
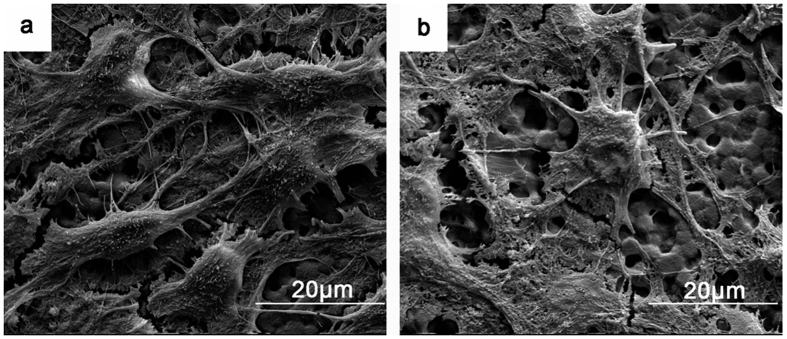
SEM images of BMSC adhesion on the PPTi (**a**) and NPTi (**b**) after incubation for 7 days. The cells spreading on the PPTi were healthy and exhibited spindle and triangular shapes with numerous amount of filopodia. Less adhesion of round cells was observed on the NPTi, and the cells exhibited a collapsed morphology. These observations indicated that the PPTi was superior for cell adhesion compared with the NPTi.

We hypothesized that the cellular behaviours on the surface of the PPTi and NPTi would differ based on their surface charges and intrinsic properties. After polarization, the dipoles of the piezoelectric PTi become oriented, resulting in the distribution of negative charges on the surface. Consequently, among the ionic/biological constituents present in the growth medium, cations adhere strongly via electrostatic interactions with the negatively charged surface. Proteins (e.g., integrin and fibronectin), and the negatively charged cytomembrane are attracted (Fig. 1). Consequently, the PPTi induced cell adhesion, proliferation and osteogenic differentiation. FTIR, DSC and XRD characterization revealed that the PPTi and NPTi displayed a majority of β-phase PVDF. The polarization process resulted in no change in the chemical composition, but a small change in the molecular structure was revealed by a small change in the phase content and a slight decrease in hydrophobicity (Fig. S3). The SKPM and zeta potential analyses of the PPTi and NPTi revealed that the PPTi surface accumulated more negative charges. PFM analysis demonstrated that the PPTi exhibited a greater piezoelectric area, whereas the NPTi exhibited weaker piezoelectric properties. The results of our cellular experiments performed *in vitro* demonstrated that the PPTi induced a more homogeneous distribution of BMSCs and osteogenic differentiation. In conclusion, the polarization process improved the surface distribution of negative charge and the piezoelectric response, resulting in inducing BMSC adhesion, proliferation and osteogenic differentiation.

In summary, an electroactive PTi was studied as a suitable material for bone regeneration applications due to its piezoelectric effect. To study the effect of the polarization of the PTi on the cellular response, BMSC adhesion, proliferation and differentiation were evaluated on the PPTi and NPTi. BMSC adhesion was influenced by the surface charge after polarization of the piezoelectric PTi. The charged PPTi induced BMSC adhesion, proliferation and osteogenic differentiation. These results confirmed that the polarization of piezoelectric film on Ti induces osteogenic differentiation and demonstrated the potential of electroactive film on Ti for cell culture and bone regeneration applications.

## Methods section

### Reagents and Materials

N, N-Dimethylformamide (DMF) and acetone were purchased from Aladdin Chem Co., Ltd, and PVDF (MV = 50000) was purchased from the Inner Mongolia 3F WanHao Fluoro Chemical Co., Ltd. Titanium (Ti) sheets for biomedical application (0.2 mm thick) according to ASTM (American Society for Testing & Materials) standard F67-2002 were obtained from the Baoji Qichen New Material Technology Co., Ltd.

### Preparation of the PTi

A PVDF membrane was prepared on the Ti sheet surface using PVDF solution (10%). In a typical procedure, PVDF was dissolved in a mixture of DMF and acetone (4:6) with stirring for 4 h at 60 °C. After cooling to room temperature, approximately 50 μL of the solution was spread on the Ti surface and dried at 80 °C for 2 h to remove the solvent via evaporation and to permit isothermal crystallization of PVDF. Electrical poling of the PVDF films was achieved through corona discharge at 100 °C. The specimen was heated from room temperature to 100 °C while the voltage applied between the electrodes was gradually increased from 0 to 1.5 kV at a rate of ~100 V/min. The sample was maintained at the elevated temperature and an applied voltage of 1.5 kV for 1 h. The sample was then cooled to room temperature (20–25 °C) under continued application of a voltage of 1.5 kV. The piezoelectric response (d33) of the poled samples was then verified using a wide-range d33 meter (YE2730A, Sinoceramics, Inc., China). The obtained piezoelectric d33 coefficient was approximately −28 pC N^−1^.

For the *in vitro* assays, circular PTi with a diameter of 10 mm was sterilized via immersion several times in 75% ethanol for 30 min. Next, the samples were washed for 5 min in sterile phosphate-buffered saline (PBS) 5 times to eliminate residual ethanol. The samples were then exposed to ultraviolet light (UV) for 30 min. The obtained samples were PPTi and NPTi.

### Characterization of the PTi

The morphology of the PTi was assessed after 120s of gold sputtering via field emission scanning electron microscopy (FESEM, Merlin, Germany) and atomic force microscopy (AFM, MFP-3D-S, Asylum Research, USA).

Attenuated total reflectance-Fourier transform infrared spectroscopy (ATR-FTIR) was performed for the PPTi and NPTi using a Bruker spectrometer (Vector-22, Switzerland, Bruker Company), from 600 to 1500 cm^−1^ at a resolution of 1 cm^−1^, to evaluate short-range molecular arrangements. The fraction of the β-phase within the crystalline region was calculated using the method first described by Osaki and Ishida^28^,^29^,^30^.

The thermal behaviour of the PTi was analysed through differential scanning calorimetry, (DSC, DSC 6, Perkin Elmer, USA). Film samples of 10 mg peeled of the PPTi and NPTi were used for each DSC measurement. The heating rate was 10 °C/min for standard DSC and 2 °C/min for modulated DSC, with a modulation period of 50 s. Melting heat enthalpy integration was performed from 60 °C to 220 °C. Before all DSC experiments, the baseline was calibrated using empty aluminium pans.

X-ray diffraction (XRD) was performed to characterize the crystallinity phase of the PTi. The samples were scanned in a 2θ range of 3 to 60 degrees. XRD was recorded with a Bruker X-ray diffractometer (Bruker D8 Advance) using Cu Kα radiation at λγ = 15.54 Å.

Piezo-force microscopy (PFM) (MFP-3D, Asylum Research, USA) images provide nanoscale insight into the distribution of polarization within PPTi by detecting its mechanical oscillation in response to the applied AC bias. To quantify the piezoelectric response of the PPTi and NPTi, a consistent AC. bias of 10 V was applied to the tip during sample scanning^31^,^32^. Piezoresponse imaging was performed over an area of 5 × 5 μm using a conductive tip with an A.C. bias of 10 V in amplitude (peak-to-peak). PFM detects tip oscillations resulting from piezoelectric vibrations of the sample surface induced by the tip-generated electric field. The PFM amplitude and phase images were recorded.

### Cell proliferation

BMSCs were cultured in Gibco Dulbecco’s Modified Eagle Medium (DMEM) supplemented with 10% foetal bovine serum (FBS) in a humidified incubator with 5% CO_2_ at 37 °C. BMSCs at passages P4-P5 were used for the experiments. The culture medium was refreshed every two days. In all experiments, the samples were sterilized with 75% ethanol and ultraviolet light. The experiments were conducted in triplicate; the number of samples was 3 for all analyses (n = 3). For BMSC culture, 500 μL of BMSC solution per well (2 × 10^4^ cells/mL) was added to PPTi and NPTi in 48-well plate. On days 1, 4 and 7, 300 μL/well of cell counting kit-8 (CCK-8) solution (0.1 mL of CCK-8 per millilitre of medium) was added, followed by incubation for 4 h at 37 °C under 5% CO_2_. Then, 200 μL of the solution from each well was transferred (in triplicate) to a 96-well plate, and the absorbance was determined at 450 nm using a microplate reader (Biocell lt2, Australia).

### Alkaline phosphatase activity

ALP is an early marker of osteogenic differentiation and is related to the production of a mineralized matrix. To determine ALP activity, BMSCs were seeded on PPTi and NPTi at a density of 2 × 10^4^ cells/mL in 500 μL of complete medium without osteogenesis induction media. After incubation for 7, 14 or 21 days, 50 μL of the cell lysate obtained after treatment with 0.2% Triton −100 at 4 °C was transferred from each well to a 96-well plate and incubated with 50 μL of p-nitrophenyl phosphate (pNPP) solution for 30 min at 37 °C. The reaction was terminated by the addition of 0.1 M NaOH solution, and the absorbance was read in a microplate reader at 405 nm.

### Osteogenesis-related gene expression

BMSCs were seeded at a density of 1 × 10^6^ cells per well and cultured for 14 days. The total RNA was extracted using the TRIzol reagent (Invitrogen). Then, 1.0 μg of RNA was reverse transcribed to obtain complementary DNA (cDNA) using the Prime Script RT reagent kit (Takara). Quantitative real-time polymerase chain reaction (qRT-PCR) was performed with SYBR Green qPCR SuperMix (Invitrogen). The sequences of the primers for the ALP, collagen I and osteopontin genes were as follows: ALP (F:5′-GTTTGCTACCTGCCTCACTT; R:5′-GAATCTGCGCAGTCTGTGT); collagen I (F:5′-CCTTCCTCAGACTTCTTTCCA; R: 5′-CTTGAATTCTCCCTCATTGG); osteopontin (F:5′-AGAGCGAGGATTCTGTGAAC; R: 5′-TCCGTAAGCCAAGCTATCAC). Gene expression was calculated via the 2^−ΔΔct^ method using Rotor-Gene Real-Time analysis software 6.0.

### Cell adhesion

At each incubation time point, the medium was removed from each well, and the samples were washed with PBS. The cells were then fixed with 4% paraformaldehyde (Aladdin) for 1 h at 4 °C. To evaluate cell morphology, the samples were washed with PBS and dehydrated in an ethanol gradient (30, 50, 60, 70, 80, 90 and 100% ethanol in water). The samples were then placed under vacuum at room temperature for 4 h. The dried samples were subsequently subjected to gold sputtering in a vacuum and evaluated via scanning electron microscopy (FESEM, Nova Nano-430, USA).

### Statistical analysis

Data are presented as the mean ± standard error of the mean; at least three replicates were averaged for each data point. Statistical analysis was performed through t-tests with Bonferroni post-hoc analysis using GraphPad Prism Software. A p-value of <0.05 was considered to indicate a significant difference and p < 0.01 was considered to indicate a very significant difference.

## Additional Information

**How to cite this article**: Zhou, Z. *et al*. Polarization of an electroactive functional film on titanium for inducing osteogenic differentiation. *Sci. Rep.* **6**, 35512; doi: 10.1038/srep35512 (2016).

## Supplementary Material

Supplementary Information
