# Supplementary material for: Polarization of an electroactive functional film on titanium for inducing osteogenic differentiation
Source: Sci Rep. 2016 Oct 20;6:35512. doi: 10.1038/srep35512 (PMC5071852; doi:10.1038/srep35512)
Supplement: Supplementary Information [file srep35512-s1.doc]

Supplementary Information

Polarization of an electroactive functional film on titanium for inducing osteogenic differentiation

Zhengnan Zhou^1,3^, Weiping Li^1,3^, Tianrui He^1,3^, Lei Qian^1,3^, Guoxin Tan^2^, Chengyun Ning^1,3*^

1 School of Materials Science and Engineering, South China University of Technology, Guangzhou 510641, China.

2 Institute of Chemical Engineering and Light Industry, Guangdong University of Technology, Guangzhou 510006, China.

3 Guangdong Key Laboratory of Biomedical Sciences and Engineering, South China University of Technology, Guangzhou 510006, China.

**Corresponding Author**

*E-mail: [imcyning@scut.edu.cn](mailto:imcyning@scut.edu.cn)


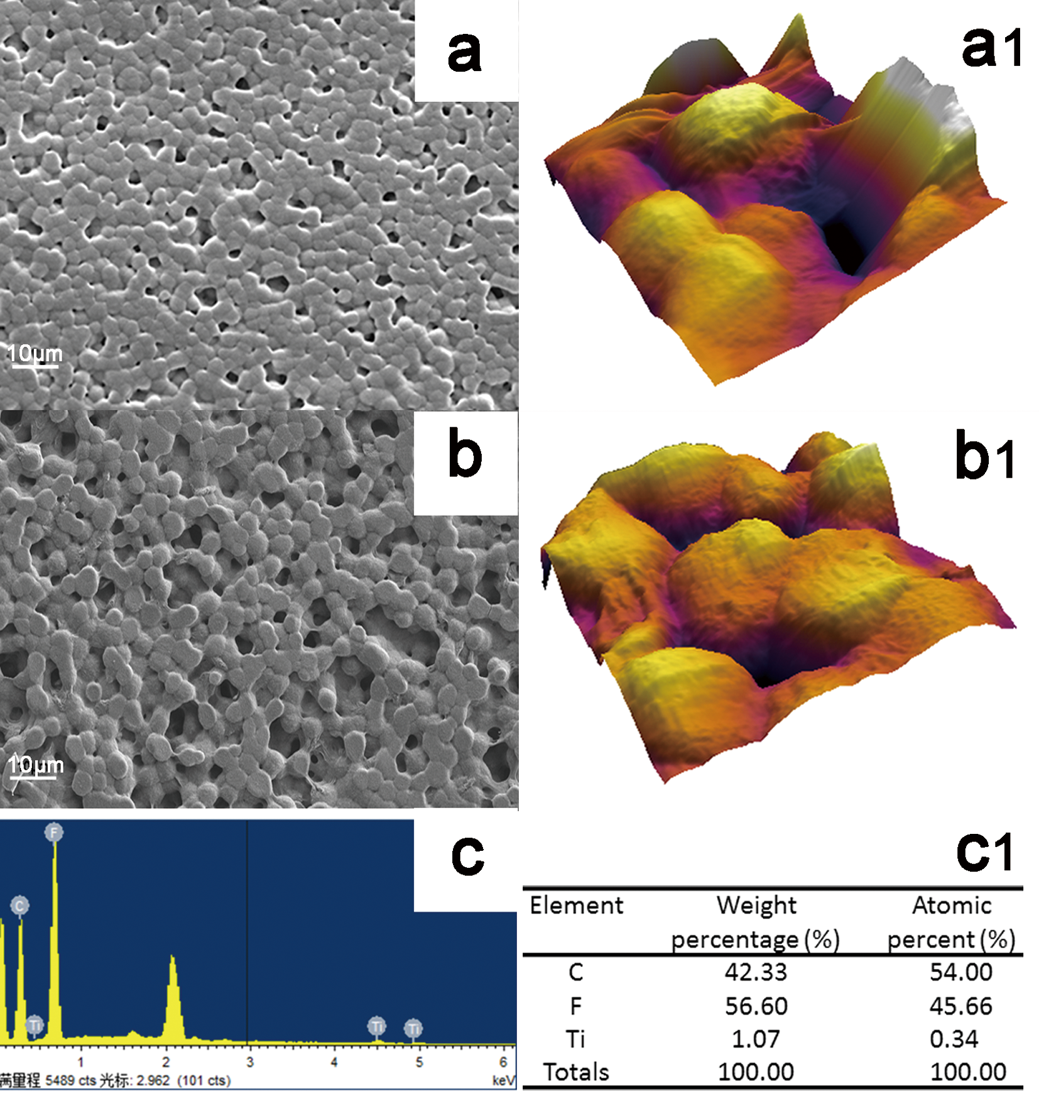


**Fig. S1.** Representative SEM images and AFM 3D topographies of the PPTi (a, a1) and NPTi (b, b1); c and c1 show the results of element energy spectrum analysis of PTi. The PPTi and NPTi were round and porous. The process of poling the PTi induced no significant differences in morphology or sample topography. Moreover, the element energy spectrum analysis demonstrated that no elemental nitrogen existed, clearly indicating that the organic solvents had been removed.


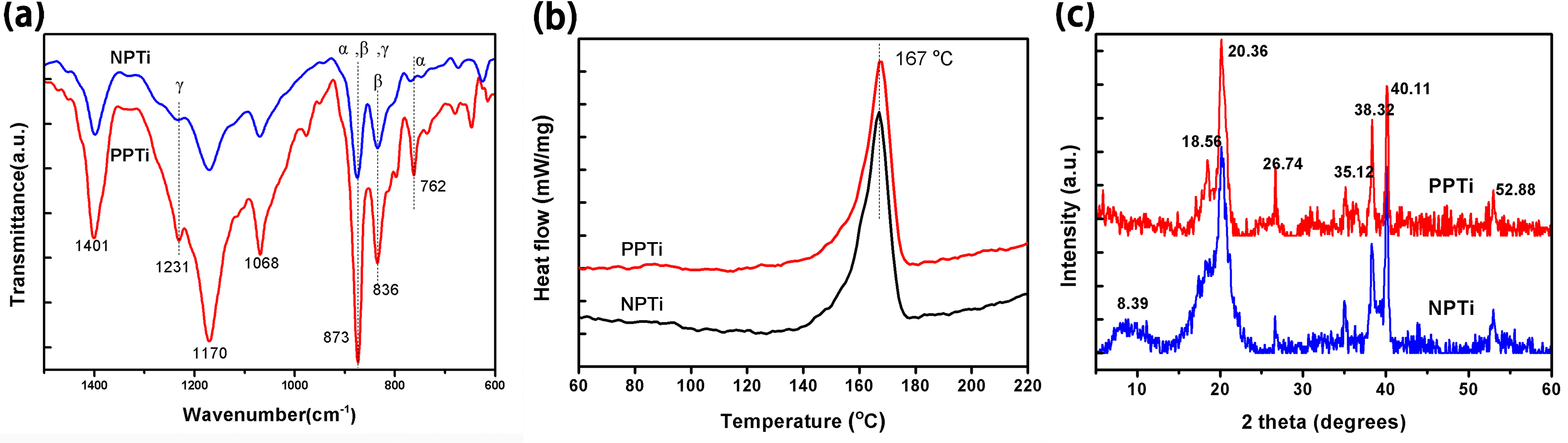


**Fig. S2.** (a) ATR-FTIR spectra of the PPTi and NPTi. (b) DSC results for the PPTi and NPTi from 60°C to 220°C with 2.0 K/min at 25°C. (c) XRD spectra of the PPTi and NPTi. The results indicate that polarization did not change the chemical composition of the PTi and that only a small change in the molecular structure and phase state occurred.


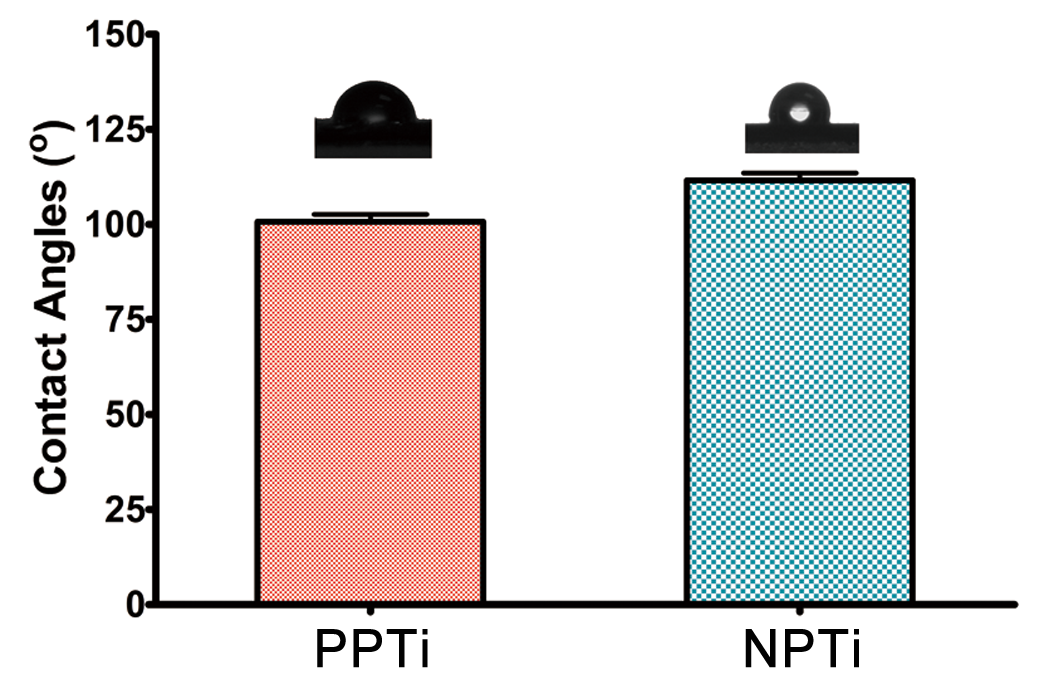


**Fig. S3.** Measurement of surface contact angles for the PPTi and NPTi. These data show that polarization of the PTi slightly decreased their hydrophobicity.
